# Cross-sectional properties of rib geometry from an adult population

**DOI:** 10.3389/fbioe.2023.1158242

**Published:** 2023-05-22

**Authors:** Sven Holcombe, Yuan Huang

**Affiliations:** International Center for Automotive Medicine, University of Michigan, Michigan Medicine, Ann Arbor, MI, United States

**Keywords:** cortical bone, rib geometry, CT, human body model (HBM), cross-sectional geometry

## Abstract

**Introduction:** Human body models (HBMs) play a key role in improving modern vehicle safety systems to protect broad populations. However, their geometry is commonly derived from single individuals chosen to meet global anthropometric targets, thus their internal anatomy may not fully represent the HBM’s target demographic. Past studies show sixth rib cross-sectional geometry differences between HBM ribs and population-derived ribs, and corrections to HBM ribs based on these data have improved HBM’s abilities to predict rib fracture locations.

**Methods:** We measure and report average and standard deviations (SDs) in rib cross-sectional geometric properties derived from live subject CT scans of 240 adults aged 18–90. Male and female results are given as functions of rib number and rib lengthwise position for ribs 2 through 11. Population means/SDs are reported for measures of rib total area, rib cortical bone area, and rib endosteal area, as well as inertial moment properties of these rib sections. These population corridors are compared between males and females, and against the baseline rib geometries defined in six current HBMs.

**Results:** Total cross-sectional area results found average males ribs to be larger than those of females by between approximately 1–2 SDs depending on rib number and position, and larger in cortical bone cross-sectional area by between 0–1 SDs. Inertial moment ratios showed female ribs being between approximately 0–1 SDs more elongated than male ribs, dependent again on rib number and position. Rib cross-sectional areas from 5 of the 6 HBMs were found to be overly large along substantial portions of most ribs when compared to average population corridors. Similarly, rib aspect ratios in HBMs deviated from average population data by up to 3 SDs in regions towards sternal rib ends.

**Discussion:** Overall, while most HBMs capture overall trends such as reductions in cross-section along shaft lengths, many also exhibit local variation that deviates from population trends. This study’s results provide the first reference values for assessing the cross-sectional geometry of human ribs across a wide range of rib levels. Results also further provide clear guidelines to improve rib geometry definitions present in current HBMs in order to better represent their target demographic.

## 1 Introduction

The thorax remains the most commonly injured body region of vehicle occupants in motor vehicle crashes, and rib fractures are the most common type of thoracic injury (Forman et al., 2019; Kullgren et al., 2020; Pipkorn et al., 2020). Finite element human body models (HBMs), such as THUMS (Shigeta et al., 2009), GHBMC (Gayzik et al., 2011), and VIVA + (John et al., 2022) are developed as numerical tools to estimate occupant kinematic and kinetic responses and injury risks via simulation. Their fidelity in indicating locations, timings, and patterns of specific injuries such as rib fractures is highly important, and research is ongoing in terms of improving definitions of rib material mechanical properties and local geometries.

Current HBMs have typically been developed to represent population-based percentiles of male or female in terms of mass and stature (e.g., 50th percentile male or 5th percentile female). As such, their specific geometry is commonly derived from a single individual meeting target population measures of anthropometry (height, BMI, chest circumstance, pelvis width, etc.), but further local and internal anatomy (e.g., bone shapes and sizes) is not strictly controlled for, and thus may not accurately represent that of the HBM’s target demographic. The biofidelity of the ribs in these HBMs needs to be evaluated and improved.

Experimental studies have shown that the cross-sectional shapes and sizes of ribs, measured local to the eventual fracture sites, have strong correlations with overall rib stiffness and force required to reach fracture (Murach et al., 2017; Agnew et al., 2018; Liebsch et al., 2021). These findings are echoed in simulation studies, with parametric rib geometry modifications finding rib cross-sectional height to be the most predictive parameter of rib strain (Iraeus et al., 2020) and that changes in overall rib cross-sectional area significantly influence rib stiffness (Fleischmann et al., 2020).

In order to build HBMs with rib geometry that represents their target demographic it is necessary to first quantify those geometric targets. With appropriate image analysis tools, medical imaging is an abundant source from which to derive these geometric targets. Previously, we used CT scans of 33 cadaveric sixth ribs to understand local bone thickness distributions and also reported average cross-sectional geometry properties within that population (Holcombe et al., 2019a). These cross-sectional data were then compared to the sixth ribs of a number of contemporary HBMs (Holcombe et al., 2020), finding that the ribs of most HBMs were substantially larger in cross-section than real ribs from their purported demographic targets. Using this information, Rampersadh et al. (2022) systematically modified the male GHBMC sixth rib model in multiple ways and found that adjusting its cross-sectional size to match the average male values reported in Holcombe et al. (2020) was the only effective method to obtain accurate predictions of fracture location during simulated loading.

Since that time, we have improved upon CT image analysis techniques to report rib cortical bone thickness from a wider range of live subject CT scans, and from rib levels 2 through 11 (Holcombe and Derstine, 2022). To date, however, the corresponding cross-sectional geometry properties of human ribs have not been expounded upon beyond just those data obtained from cadaveric sixth ribs. In this study we provide these missing cross-sectional data, and further evaluate the extent to which currently available HBM ribs are indeed representative of the real rib geometries from their constituent demographics.

## 2 Methods

### 2.1 Study population

Ribs from a total of 240 chest CT scans were used for this study under IRB HUM00041441 as described in detail previously in (Holcombe and Derstine, 2022). The population consisted of approximately uniform counts (15 or greater) of males and females within each decade of age between 20 and 90 years, with demographic averages for height, weight, and BMI provided in Table 1. Subjects were all scanned at a Midwestern United States medical facility (Michigan Medicine) and reflect the demographics of that region [see (Holcombe and Derstine, 2022) for further details]. Scans were obtained using a standard soft tissue reconstruction kernel at 0.625 mm slice spacing, with axial resolution ranging between 0.50 mm/px and 0.97 mm/px. Subjects had normal thoracic skeletal anatomy, and ribs identified as having one or more fractures were excluded from the study. A total of 4,014 ribs numbered 2 through 11 were thus included.

**TABLE 1:** CT subject demographics by sex (average ± SD).

Sex	n	Age (years)	Wt. (kg)	Ht. (cm)	BMI
Male	118	55 ± 19	89 ± 19	177 ± 8	28.3 ± 5.4
Female	122	54 ± 19	80 ± 22	163 ± 8	30.3 ± 8.1

### 2.2 Image processing and cross-sectional measurements

In previous work (Holcombe and Derstine, 2022), 3D curves were placed along each rib within the CT scans, and small reformatted cross-sectional image patches were then created normal to these curves at successive increments of 2.5% of the rib’s overall length. Each sectional patch was processed using a cortical bone mapping (CBM) algorithm to identify outer periosteal borders and inner endosteal borders of the rib’s cortical bone.

In the current study we measure and report the cross-sectional area and inertial moment properties from the shapes created by these cortical bone borders. The measurements consist of the total sub-periosteal area (Tt.Ar), the cortical bone area (Ct.Ar), the endosteal area (Es.Ar), and the cortical shell’s maximal (or principal) and minimal (or secondary) area inertial moments (Imax and Imin). A derived ratio of these moments (the log transform of Imax divided by Imin), Irat, is also created.

Results for each cross-sectional property were pooled by sex, rib number, and rib position (lengthwise along the rib), and average and standard deviations in these property distributions are reported.

### 2.3 HBM rib geometry

The same cross-sectional rib measurements were obtained from all ribs of 6 human body models that are currently use within the automotive safety community, listed in Table 2. The process to extract these measurements [described in greater detail in Holcombe et al. (2020)] took successive circumferential rings formed by the nodes of shell elements representing the cortical bone for each model’s ribs. Periosteal and endosteal rib borders were defined by offsetting each ring of rib nodes by the shell thickness values defined within each HBM rib. Cross-sectional properties of these borders were measured equivalently to those obtained from live subject CTs and comparisons made to the average ±1SD corridors as a function of rib number and rib position from all live subjects of the equivalent sex.

**TABLE 2:** Models presented in this study along with their specific subject or target demographics.

Model	Ver	Sex	%ile	Age	Wt.(kg)	Ht.(cm)	BMI	Ref
GHBMC M50	5.0	M	50th	26	78.0	174.9	25.7	GHBMC, (2019)
THUMS M50	4.0	M	50th	39	77.0	173.0	25.7	Toyota Motor Corporation, (2011b)
VIVA + M50	1.0	M	50th	50	76.8	175.3	25.0	John et al. (2022)
GHBMC F05	6.0	F	5th	24	48.1	149.9	21.4	Davis et al. (2016)
THUMS F05	4.0	F	5th	38	52.0	154.0	21.9	Toyota Motor Corporation, (2011a)
VIVA + F50	1.0	F	50th	50	62.7	161.6	24.0	John et al. (2022)

**FIGURE 1:**
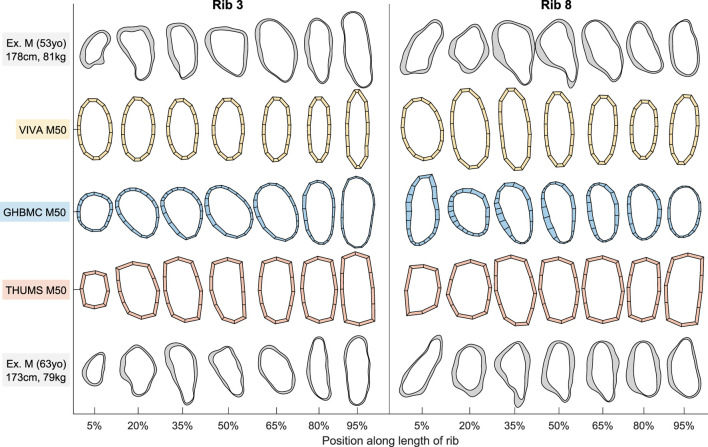
Male HBM rib cross-sections from ribs 3 and 8 alongside exemplar male subject cross-sections. North/east/south/west sides of each section correspond to rib superior/cutaneous/inferior/pleural aspects, respectively.

**FIGURE 2:**
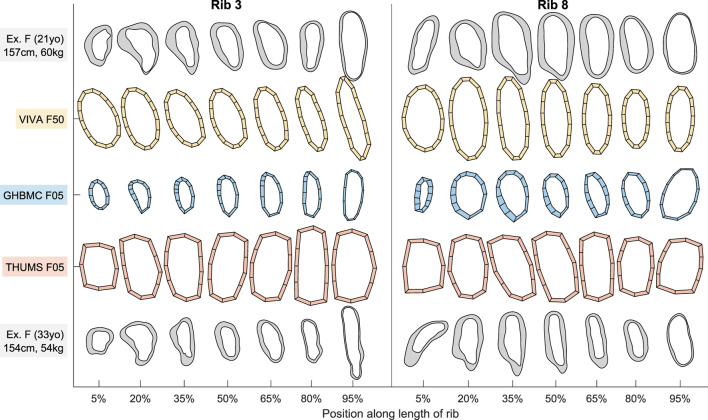
Female HBM rib cross-sections from ribs 3 and 8 alongside exemplar female subject cross-sections. North/east/south/west sides of each section correspond to rib superior/cutaneous/inferior/pleural aspects, respectively.

Sample cross-sectional geometry from ribs 3 and 8 of the VIVA + M50, GHBMC M50, and THUMS M50 HBMs are shown in Figure 1 alongside exemplar rib sections taken from two live subjects matching the 50th percentile stature and weight criteria for these models. Figure 2 similarly shows VIVA + F50, GHBMC F05, and THUMS F05 HBM rib sections along with an exemplar subject from the 50th and 5th percentile demographic ranges. As seen in Figures 1, 2, the VIVA + and GHBMC models each utilize 16 shell elements around each rib circumference whereas the THUMS models are comprised of 12 elements. The GHBMC models each implement a cortical bone thickness that varies across the surface of each rib, while the THUMS and VIVA + models each implement a uniform cortical bone thickness of 0.7 mm across all ribs.

## 3 Results

### 3.1 Male and female results

**FIGURE 3:**
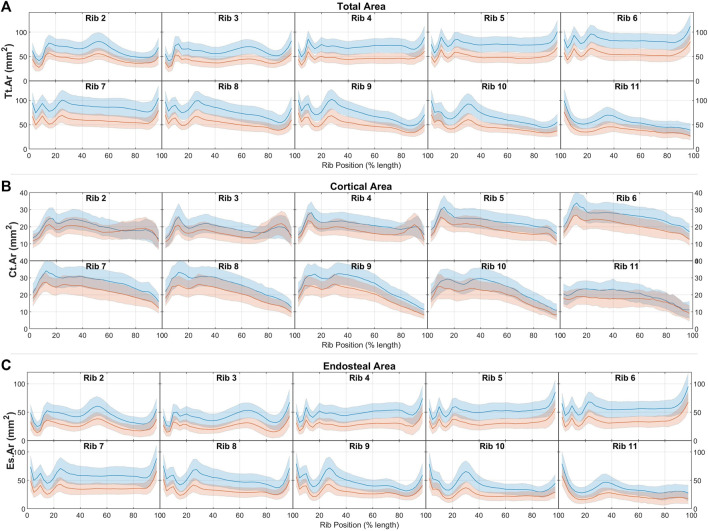
Male (blue) and female (red) rib cross-sectional area properties of **(A)** total area, **(B)** cortical bone region area, and **(C)** endosteal area, given as average ± 1SD corridors.

Male and female average cross-sectional area measurements (Tt.Ar, Ct. Ar, and Es.Ar) of ribs 2 through 11 are summarized by rib number and position along the rib in Figure 3 (a, b, and c, respectively). These average and standard deviation data are also provided as Supplementary Material. In Figure 3 we see consistent shared patterns in each of these area measurements between the two sexes, but with males having generally larger rib sections by between 2 SDs (Tt.Ar and Es.Ar) and 1 SDs (Ct.Ar) as compared to females. These differences are consistently seen across all rib levels and regions with the exception of the sternal ends of ribs 2-4, whereby the difference in Ct.Ar diminishes towards parity at between 70% and 90% of the rib’s length.

**FIGURE 4:**
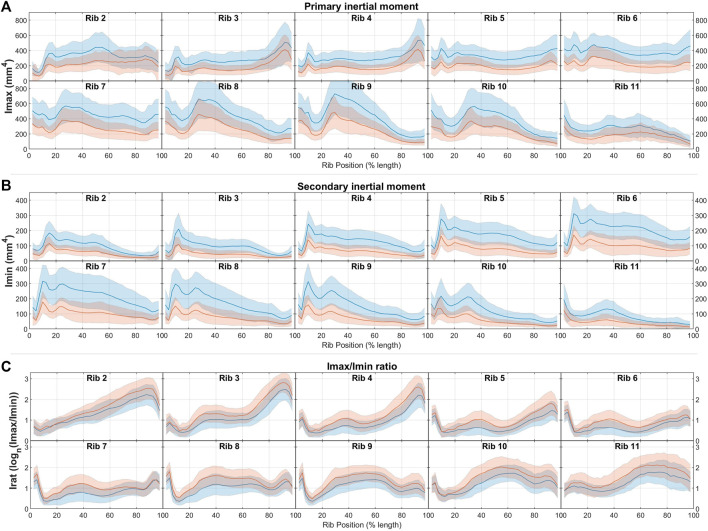
Male (blue) and female (red) rib cross-sectional inertial moment properties of **(A)** total area, **(B)** cortical bone region area, and **(C)** endosteal area, given as average ± 1SD corridors.

Comparisons of area inertial moment measures between males and females are presented in Figure 4. In line with having larger total areas, males have similarly larger principal (Imax) and secondary inertial moments (Imin) than females. This difference is found across all ribs and all rib locations, and ranges between approximately 1 and 2 standard deviations at different regions across the rib cage. Female ribs are also seen to have higher Irat ratios (i.e., have more elongated rib cross-sections) by a margin of less than 1 SD across most rib regions.

### 3.2 Male HBM comparisons

**FIGURE 5:**
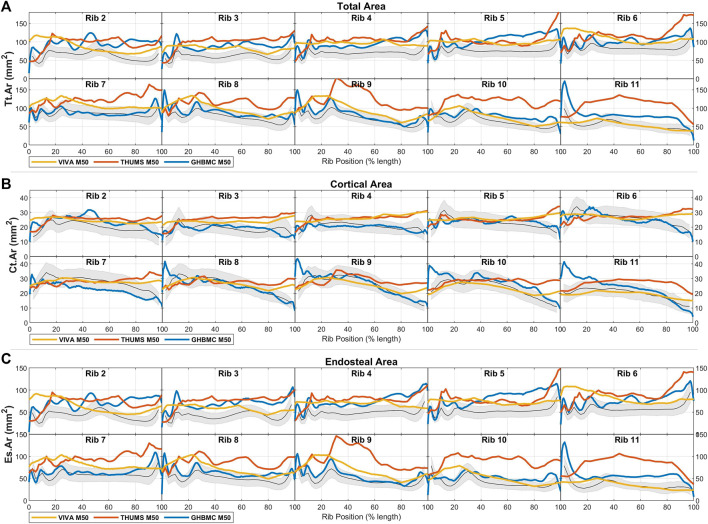
Male HBM rib cross-sectional area properties for **(A)** total area, **(B)** cortical bone region area, and **(C)** endosteal area, each shown compared to average ± 1SD corridors (gray region) from male live subjects.

**FIGURE 6:**
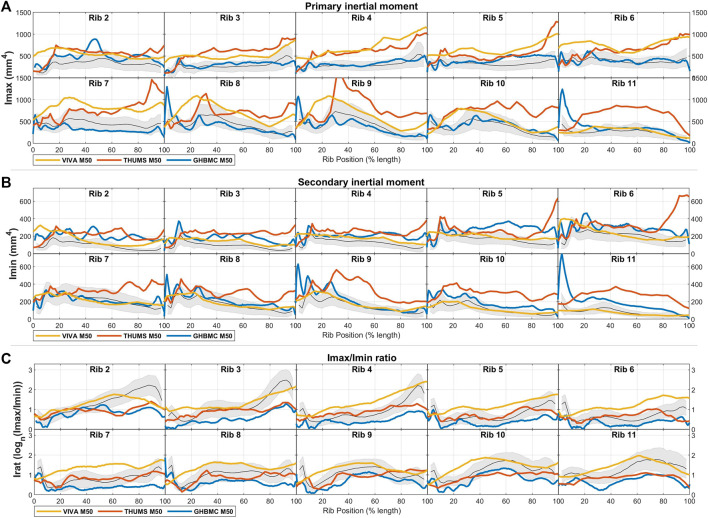
Male HBM rib cross-sectional area properties for **(A)** principal inertial moment, **(B)** secondary inertial moment, and **(C)** inertial moment ratio, each shown compared to average ± 1SD corridors (gray region) from male live subjects.

Rib geometry measurements taken from the three male HBMs (GHBMC M50, THUMS M50, and VIVA + M50) are shown superimposed over the average male corridors for those same measures in Figures 5, 6. Figure 5 shows that, in general, the male HBMs tend to each have larger overall rib cross-sections (Tt.Ar and Es.Ar measures) than the male population average. Correspondences between the HBM rib sizes and the population corridors vary greatly by rib and by local region. Table 3 quantifies these comparisons (for all cross sectional properties within the GHBMC M50 and other models as described further below) in terms of the percentage length of each rib that falls within (or above or below) 1SD from average results found within the population.

**TABLE 3:** Comparisons by rib between 50th percentile HBMs and male or female rib cross-sectional property population data. Each property lists the discrepancy along the rib’s length between the model and population expectations, along with the percentage of the rib’s length above (high), within (in), or below (low) 1SD from the population average.

		THUMS M50				GHBMC M50				VIVA + F50				VIVA + M50			
		Avg	high	in	low	Avg	high	in	low	Avg	high	in	low	Avg	high	in	low
		SDs	%	%	%	SDs	%	%	%	SDs	%	%	%	SDs	%	%	%
Rib 2	Tt.Ar	+2.5	92	8	0	+2.6	92	8	0	+2.7	87	13	0	+1.9	69	31	0
	Ct.Ar	+1.0	49	51	0	+0.8	28	72	0	+0.9	36	64	0	+0.9	28	72	0
	Es.Ar	+2.3	95	3	3	+2.4	85	15	0	+2.5	79	21	0	+1.7	64	36	0
	Imax	+1.6	74	26	0	+1.2	56	44	0	+1.8	74	26	0	+2.0	59	41	0
	Imin	+3.4	90	10	0	+3.3	90	10	0	+2.8	79	21	0	+2.0	62	38	0
	Irat	−0.9	0	56	44	−1.2	0	51	49	−0.3	0	82	18	+0.0	5	77	18
Rib 3	Tt.Ar	+2.9	90	10	0	+2.0	92	8	0	+2.5	97	3	0	+1.5	69	31	0
	Ct.Ar	+1.4	67	33	0	+0.1	0	100	0	+1.1	49	51	0	+1.0	44	56	0
	Es.Ar	+2.5	92	5	3	+2.1	95	5	0	+2.3	87	13	0	+1.3	67	33	0
	Imax	+2.4	87	13	0	+0.2	0	100	0	+2.3	85	15	0	+1.8	87	13	0
	Imin	+4.2	87	13	0	+2.3	79	21	0	+2.6	95	5	0	+1.4	62	38	0
	Irat	−0.5	8	62	31	−1.5	0	10	90	−0.2	10	74	15	+0.5	18	79	3
Rib 4	Tt.Ar	+2.4	95	5	0	+1.7	92	8	0	+3.1	97	3	0	+1.9	92	8	0
	Ct.Ar	+1.1	51	49	0	−0.2	3	95	3	+1.4	67	33	0	+1.2	49	51	0
	Es.Ar	+2.2	95	5	0	+1.9	100	0	0	+2.8	92	8	0	+1.6	79	21	0
	Imax	+2.6	92	8	0	+0.2	0	100	0	+4.4	100	0	0	+3.4	95	5	0
	Imin	+2.4	95	5	0	+1.6	59	41	0	+2.2	97	3	0	+1.0	31	69	0
	Irat	+0.1	10	72	18	−1.3	0	33	67	+0.7	44	54	3	+1.4	77	21	3
Rib 5	Tt.Ar	+1.8	97	3	0	+1.9	77	23	0	+2.8	97	3	0	+1.6	92	8	0
	Ct.Ar	+0.6	21	79	0	+0.2	3	92	5	+1.2	46	54	0	+0.7	31	69	0
	Es.Ar	+1.8	97	3	0	+2.1	79	21	0	+2.8	92	8	0	+1.5	87	13	0
	Imax	+1.7	74	26	0	+0.7	36	64	0	+3.8	100	0	0	+2.5	95	5	0
	Imin	+1.6	54	46	0	+2.0	72	28	0	+1.6	77	23	0	+0.6	21	79	0
	Irat	+0.1	8	79	13	−1.1	0	36	64	+0.8	49	46	5	+1.5	79	18	3
Rib 6	Tt.Ar	+2.1	85	15	0	+1.2	59	41	0	+2.6	95	5	0	+1.7	77	23	0
	Ct.Ar	+0.4	21	74	5	−0.1	3	97	0	+0.9	28	72	0	+0.4	23	77	0
	Es.Ar	+2.3	92	8	0	+1.4	67	33	0	+2.8	92	8	0	+1.8	79	21	0
	Imax	+1.8	77	23	0	+0.1	0	100	0	+3.2	100	0	0	+2.5	100	0	0
	Imin	+1.9	54	46	0	+1.2	64	36	0	+1.4	41	59	0	+0.4	10	90	0
	Irat	+0.0	23	62	15	−1.2	0	26	74	+1.0	56	38	5	+1.8	92	3	5
Rib 7	Tt.Ar	+2.0	87	13	0	+0.1	5	95	0	+2.0	95	5	0	+1.3	64	36	0
	Ct.Ar	+0.3	21	77	3	−0.8	3	54	44	+0.6	21	79	0	+0.2	13	87	0
	Es.Ar	+2.3	90	10	0	+0.4	18	82	0	+2.3	92	5	3	+1.5	72	28	0
	Imax	+1.8	72	28	0	−0.8	0	79	21	+2.5	97	3	0	+2.2	95	5	0
	Imin	+1.3	38	62	0	+0.1	5	95	0	+0.8	28	72	0	+0.1	5	95	0
	Irat	+0.0	5	90	5	−1.2	0	49	51	+1.1	54	41	5	+1.7	87	8	5
Rib 8	Tt.Ar	+2.2	92	8	0	+0.7	26	74	0	+2.2	92	8	0	+1.3	54	46	0
	Ct.Ar	+0.5	23	77	0	−0.1	5	95	0	+0.7	18	82	0	+0.3	13	87	0
	Es.Ar	+2.4	92	8	0	+0.8	26	74	0	+2.4	87	13	0	+1.4	72	28	0
	Imax	+1.7	59	41	0	−0.2	3	97	0	+2.2	95	5	0	+1.7	87	13	0
	Imin	+1.7	67	33	0	+0.6	18	82	0	+1.2	46	54	0	+0.3	8	92	0
	Irat	−0.4	0	90	10	−1.1	0	44	56	+0.3	13	82	5	+1.0	49	46	5
Rib 9	Tt.Ar	+3.6	95	5	0	+0.6	26	74	0	+2.7	97	3	0	+1.5	82	18	0
	Ct.Ar	+0.9	31	69	0	+0.2	5	95	0	+1.1	31	69	0	+0.2	13	87	0
	Es.Ar	+3.9	95	5	0	+0.6	21	79	0	+2.9	95	5	0	+1.8	82	18	0
	Imax	+2.9	79	21	0	−0.2	3	92	5	+3.1	92	8	0	+1.6	51	49	0
	Imin	+2.8	95	5	0	+0.8	28	72	0	+1.8	90	10	0	+0.5	23	77	0
	Irat	−0.3	8	67	26	−1.0	0	46	54	+0.3	15	79	5	+0.7	26	69	5
Rib 10	Tt.Ar	+4.0	92	8	0	+1.1	62	38	0	+2.0	85	15	0	+0.8	15	82	3
	Ct.Ar	+1.0	36	64	0	+0.8	21	79	0	+0.7	23	77	0	+0.1	13	87	0
	Es.Ar	+4.4	95	5	0	+1.1	64	36	0	+2.1	69	31	0	+0.9	44	54	3
	Imax	+3.3	87	13	0	+0.4	13	87	0	+2.0	85	15	0	+1.0	36	62	3
	Imin	+4.0	92	8	0	+1.4	72	28	0	+1.2	62	38	0	+0.1	3	97	0
	Irat	−0.8	0	64	36	−1.1	0	38	62	+0.2	21	74	5	+0.7	36	59	5
Rib 11	Tt.Ar	+4.7	92	8	0	+2.0	82	18	0	+0.7	23	74	3	+0.0	10	87	3
	Ct.Ar	+1.4	69	31	0	+0.8	28	72	0	+0.1	3	97	0	−0.1	3	97	0
	Es.Ar	+4.6	92	5	3	+1.9	85	15	0	+0.7	23	74	3	+0.1	15	82	3
	Imax	+3.8	90	10	0	+0.9	28	72	0	+0.4	18	82	0	+0.3	10	90	0
	Imin	+5.4	95	5	0	+2.4	90	10	0	+0.6	21	79	0	−0.2	0	100	0
	Irat	−0.9	0	54	46	−1.4	0	8	92	−0.3	0	97	3	+0.4	28	72	0

Comparing to population average values, the GHBMC M50 model ribs 2 through 5 are generally larger in Tt.Ar than average male ribs (over 75% of these ribs’ length beyond 1SD above population averages), with lower ribs 7 through 11 falling near to or within 1 SD of male average values. Measured cortical bone content (Ct.Ar) matches population values more closely, falling within 1SD of expected average values along approximately 90% of all GHBMC M50 ribs.

The THUMS M50 model ribs are uniformly larger in cross-section than average male ribs, with greatest discrepancy in Tt.Ar from average male values of over 4 SDs for portions of ribs 9 through 11. With their uniform bone thickness, the THUMS M50’s Ct.Ar measures are relatively uniform across all ribs, leading to largest discrepancies with population Ct.Ar data towards the sternal ends of ribs 4–11.

The VIVA + M50 model ribs are larger in Tt.Ar than average male data by approximately 1.5SDs across most ribs, and track population averages in Ct.Ar more closely on most rib regions. The greatest discrepancies in Ct.Ar tend to be seen at the sternal ends of all ribs.

Comparisons of the area inertial moments of these male HBM ribs to average male population data are shown in Figure 6 and again quantified in Table 3. Results here show the primary and secondary inertial moments (Imax, Imin) being larger than expected for the THUMS M50 model across most ribs. This is true also for Imax in the VIVA + M50 model across ribs 2 through 9, but Imin values fall much closer to population averages across all ribs. Inertial moment discrepancies are most prominent when considering Irat, which reaches a ratio of over 10-to-1 towards the sternal end of average adult ribs, whereas corresponding regions of the HBMs reach only ratios of 4-to-1 in similar regions.

### 3.3 Female HBM comparisons

**FIGURE 7:**
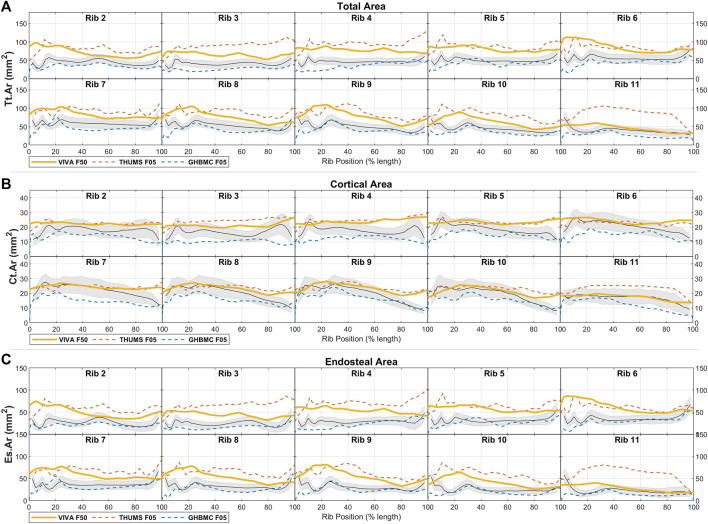
Female HBM rib cross-sectional area properties for **(A)** total area, **(B)** cortical bone region area, and **(C)** endosteal area, each shown compared to average ± 1SD corridors (gray region) from female live subjects.

Cross-sectional area comparisons between female HBM ribs and female population average measurements are shown in Figure 7. Here, the VIVA + F50 model is expected to represent a 50th percentile individual in stature and weight, whereas the GHBMC F05 and THUMS F05 models represent 5th percentile individuals. Correspondingly only the VIVA + F50 is included in quantified comparisons given in Table 3. The THUMS F05 and VIVA + F50 models are each seen to have consistently larger cross-sectional area (Tt.Ar) than average female ribs. With uniform cortical bone thickness definitions of 0.7 mm, these models also tend to have rib regions with greater Ct. Ar than a female population average, particularly in central regions of ribs 2-4 and towards the sternal ends of ribs 5–9. The GHBMC F05 model, on the other hand, is generally smaller in both Tt. Ar and Ct. Ar than an average female cohort by approximately 1SD for most ribs.

**FIGURE 8:**
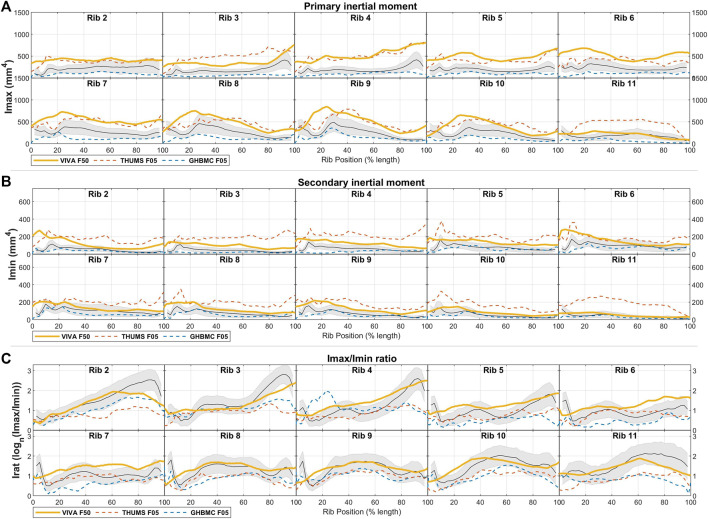
Female HBM rib cross-sectional area properties for **(A)** principal inertial moment, **(B)** secondary inertial moment, and **(C)** inertial moment ratio, each shown compared to average ± 1SD corridors (gray region) from female live subjects.

Area inertial moment comparisons between average population data and female HBMs are shown in Figure 8. These generally follow trends of Imax and Imin inertial measures being larger than average female ribs for THUMS F05 and VIVA + F50 models, and Imax being smaller than average ribs for the GHBMC F05 model.

Taken together, the ratio of inertial moments (Irat) is generally lower than average population values for the THUMS F05 and GHBMC F05 models. Like for the male models, this discrepancy is particularly prominent towards the sternal end of ribs 2-4 which is highly elongated in females up to an average ratio of 18-to-1. Notably the VIVA + F50 model does stay primarily within a 1SD corridor across all rib regions, and reaches an Irat ratio of 10-to-1 at these elongated regions of ribs 2-4.

## 4 Discussion

In this study we report average rib cross-sectional properties from a wide range of adult CT scans at ribs 2 through 11. These data, along with population variance, are provided as functions of local rib locations, and we compare a series of currently used finite element HBM ribs to this real-world rib data.

In general, all HBMs considered in this study with the exception of the GHBMC F05 tended to have overly large cross-sections to most of their ribs. Ribs from the GHBMC M50 were next closest to average population sizes, with Tt.Ar from ribs 7 through 9 and Ct.Ar from all ribs falling within a 1SD corridor of population values for 75% or more of those ribs’ lengths. In other models no two consecutive ribs met this criteria for Tt.Ar, and values for Ct.Ar consistently exceeded the 1SD corridors towards the sternal ends of all ribs.

The underlying cause of oversized rib Tt.Ar has been previously identified and discussed in depth (Holcombe et al., 2019b). In that study it was found that traditional threshold-based segmentation routines (which were likely used when establishing rib geometries for all of the HBM evaluated here) can overestimate the positions of periosteal rib borders by an average of 0.42 mm and thus produce cross-sectional dilation in Tt.Ar of 35%. A remedy to this dilation of thin bone structures is the use of cortical bone mapping segmentation algorithms designed to more accurately detect true bone boundaries on CT images (Treece and Gee, 2015; Holcombe et al., 2018; Holcombe et al., 2019a; Holcombe and Derstine, 2022).

Another important aspect of rib mechanics is the cross-sectional shape which, here, has been characterized in terms of its aspect ratio by using the log ratio (Irat) of the two inertial moments, Imax and Imin. Population results here found a distinct elongation of ribs 2 through 5 towards their sternal ends producing a peak in Irat at 90% along the rib’s length of up to log_*n*_15 in males and log_*n*_18 in females (i.e., respective Imax magnitudes 15 and 18 times greater than Imin). Most HBMs failed to reproduce this characteristic rib shape, with only the VIVA + models matched this aspect ratio along parts of their upper ribs, albeit with their Irat magnitudes increasing somewhat linearly along the whole rib’s shaft rather than following the exact change in ratio that was expected from population data. Taken together, these differences in Tt.Ar and Irat indicate that most HBM ribs can be overly large and overly round towards their sternal ends - a region in which rib fractures commonly occur (Lee et al., 2015).

HBMs have traditionally had trouble reproducing the timing and the onset of rib fractures seen in mechanical or field studies (Schoell et al., 2015), however these issues have been improved when simulating more detailed and personalized rib models rather than generic HBM ribs (Iraeus et al., 2019). Importantly, Rampersadh et al. (2022) also showed that even generic HBM sixth ribs can report accurate fracture locations if they are first modified to meet population average values in terms of local cross-sectional size and aspect ratio. For this reason, this study aims firstly to make these population data available for ribs 2 through 11 such that similar HBM modifications might be made to those ribs also. A secondary goal of this study is to provide context to researchers regarding the mechanical makeup of common HBM ribs and how they compare to such population data. It should be noted, however, that rib cross-sectional geometry is not the only factor influencing rib fracture risk during loading. Other factors such as bone material, thickness, and failure properties should similarly be considered, particularly if geometric modifications are made to previously validated HBMs.

When interpreting the population corridors presented here it is important to understand the differences between the demographic target represented by each HBM and the included subjects for each given corridor. Firstly, each HBM targets a specific demographic cohort (average sized males and females in both height and weight) whereas the data presented here is average across the full male or female sampled population. This choice was made for a number of reasons. Firstly, true population variance in these specific measures is useful to quantify, and this would be lost if reporting only variance within the smaller subset. Secondly, inspection of measurements obtained from only subjects meeting average height and weight criteria found that their average values were highly similar to whole population averages. And finally, the inclusion of all subjects reduces the influence that any given outlier individual may have on overall average values.

Despite providing cross-sectional data from the largest number of ribs and population available to date, the current study does not include data from rib 1 or rib 12 since each of these were excluded from the original cortical bone thickness study. The first rib has greater mechanical significance in most blunt trauma scenarios and efforts should be taken to characterize its shape and cross-sectional properties. Furthermore, the included population is sourced from a Midwestern United States adult population thus care should be taken when applying results to subjects from differing regions.

## 5 Conclusion

This study has derived sex-specific corridors of cross-sectional geometric measurements along lengths of ribs 2 through 11 from an adult population of 240 subjects and over 4,000 ribs. Through comparisons to current HBMs we have identified key geometric differences that are likely to affect each models’ mechanical behavior. The reference data provided within this study can be used to alter these HBM ribs and improve their fracture prediction capabilities.

## Data Availability

The original contributions presented in the study are included in the article/Supplementary Material, further inquiries can be directed to the corresponding author.
